# Tobacco Users among the Patients who Visited Dental Outpatient Department of a Tertiary Care Hospital: A Descriptive Cross-sectional Study

**DOI:** 10.31729/jnma.6596

**Published:** 2021-10-31

**Authors:** Rosina Bhattarai, Santosh Adhikari

**Affiliations:** 1 Department of Community Dentistry, College of Medical Sciences, Bharatpur, Nepal

**Keywords:** *dental caries*, *periodontal disease*, *tobacco*

## Abstract

**Introduction:**

Smoked and smokeless forms of tobacco is a preventable cause of morbidity and mortality with inevitable effects on the oral cavity as well. The aim of the study was to find out the prevalence of tobacco users among the patients who visited dental outpatient departments of a tertiary care hospital.

**Methods:**

A descriptive cross-sectional study was carried out among 255 patients visiting the dental outpatient department of a tertiary care hospital over the duration of six months. Data collection was done through personal interviews and clinical examinations. History of tobacco use was taken and patients were divided into current users, never users and ever users. Clinical examination was done using Decayed Missing Filled Teeth index, community periodontal index modified and loss of attachment. Point estimate at 95% confidence interval was calculated along with frequency and proportion for binary data.

**Results:**

Among 255 patients, 91 (35.69%) (95% Confidence Interval = 29.81-41.57) patients used tobacco. Among them, 72 (28.2%) were current users, who had taken tobacco at least once during the past 30 days and 19 (7.5%) were ever users who had taken tobacco but not during the past 30 days.

**Conclusions:**

The prevalence of tobacco use among our study participants is similar in comparison to that of the general population. Our study shows greater prevalence of the use of a smokeless form of tobacco than the smoked form of tobacco.

## INTRODUCTION

Smoked and smokeless form of tobacco is one of the leading global preventable causes of mortality and morbidity worldwide. Cigarette has been considered as a cause for health inequalities.^1^ Massive use of tobacco is considered a global epidemic reducing family's finances for basic needs and affecting economic development. More than 10% of total household expenditures are spent on tobacco in households of low and middle-income countries.^2^

Tobacco consumption in both forms is highly prevalent in Nepal. The national-level non-communicable disease risk factor survey (STEPS) conducted in 2008 showed that the prevalence of consumption of smokeless tobacco among individuals of age 15-64 years was 18.5% with inevitable effects on the oral cavity.^3^ No studies have been carried out about tobacco use and its effect on oral health in Chitwan.

The aim of the study was to find out the prevalence of tobacco users among the patient who visited the dental outpatient departments of a tertiary care hospital.

## METHODS

A descriptive, cross-sectional study was carried out among 255 adult population attending dental OPD of College of Medical Sciences, Bharatpur over a span of six months from October 2020 to March 2021. Ethical clearance was given by the College of Medical Sciences and Teaching Hospital - Institutional Review Committee (COMSTH-IRC) (Reference number: 2020059). Informed consent was taken from the participants prior to the study. Inclusion criteria included those who are residents of Chitwan, adults greater than 18 years of age and those who visited the dental outpatient department of the College of Medical Sciences and Teaching Hospital and are willing to participate in the study. Individuals not giving consent for the study, physically challenged and mentally compromised elderly people with cognitive impairment and elderly people with terminal illness were excluded from the study. A convenience sampling technique was used and the sample size was calculated with the following formula,

n = Z^2^ × p × q / e^2^

  = (1.96)^2^ × 0.185 × 0.815 / (0.05)^2^

  = 232

where,

n = sample size
Z = 1.96 at 95% confidence interval
p = prevalence rate of tobacco use according to national level non-communicable disease risk factor survey which was 18.5%,^6^
q = 1-p
e = margin of error, 5%.

Hence, the sample size was calculated to be 232. Taking 10% as a non-response rate i.e. 23, the total sample size was calculated to be 255.

Data collection was done through personal interviews and clinical examinations. The personal interview consisted of information on name, age, sex and tobacco history. Tobacco users were classified as current users-who had used tobacco once or more during the previous 30 days, ever-users-who have tried tobacco but not during the previous 30 days and never-users - who did not consume tobacco.^4^ Clinical examinations consisted of DMFT index, community periodontal index (CPI) modified and loss of attachment according to WHO Oral Health Survey, Basic methods, 5^th^ edition using mouth mirror and CPI probe.^5^ For the assessment of periodontal status, the highest score of the patient for gingival bleeding and the periodontal pocket was noted. The highest score for the loss of attachment was also noted.

Data entry was done in Microsoft Excel Sheet and statistical analysis was done using Statistical Package for Social Sciences Version 20. Point estimate at 95% confidence interval and descriptive statistics were calculated.

## RESULTS

Among 255 patients, 91 (35.69%) (95% CI = 29.8141.57) patients used tobacco. Among them, 72 (28.2%) were current users, who have taken tobacco at least once during the past 30 days and 19 (7.5%) were ever users who have taken tobacco but not during the past 30 days.

The majority of the current users i.e. 42 (58.3%) belonged to the age group of 35-64 years old and the majority of the never-users i.e. 106 (64.6%) belonged to the age group of 18-34 years. Our study showed that 56 (77.8%) were current users (Table 1).

**Table 1:** Demographic characteristics of the population.

Variables		Current users n (%)	Ever users n (%)	Never users n (%)
Age in years	18 - 34	14 (19.4)	5 (26.3)	106 (64.6)
	35 - 64	42 (58.3)	9 (47.4)	49 (29.9)
	>65	16 (22.2)	5 (26.3)	9 (5.5)
Gender	Male	56 (77.8)	13 (68.4)	68 (41.5)
	Female	16 (22.2)	6 (31.6)	96 (58.5)
Total		72	19	164

The age of the patients ranged from 18-80 years with the mean age of the participants being 38.43 years. The majority of the participants i.e. 125 (49%) them belonged to the age group of 18-34 years. 100 (39.2%) of them belonged to the age group of 35-64 years while 30 (11.8%) of them were 65 years or older. Out of the total participants, 137 (53.7%) were males and 118 (46.3%) of them were females.

Among all the current tobacco users, it was found that 25 (34.7%) of them consumed cigarettes, 32 (44.4%) of them consumed smokeless forms of tobacco and 15 (20.8%) of them consumed both cigarette and smokeless tobacco.

Mean DMFT was found to be highest among the current users i.e. 3.98±3.18 with mean decayed teeth 2.74, missing teeth 1.10±2.07. However, mean filled teeth was higher among ever user group i.e. 0.53 ±1.12 (Table 2).

**Table 2:** Decayed, Missing, Filled teeth among different groups of tobacco users.

Groups	Decayed (D)	Missing (M)	Filled (F)	DMFT
	Mean±SD	Mean±SD	Mean±SD	Mean±SD
Current user	2.74±2.27	1.10±2.07	0.15±0.70	3.98±3.18
Ever user	1.68±1.76	0.26±0.65	0.53±1.12	2.47±2.01
Never user	2.20±1.97	0.40±1.01	0.42±1.09	3.01±2.63

Oral mucosal lesions were present in 13 (5.1%) participants. Gingival bleeding was found to be absent in the majority of current users i.e. 38 (52.8%) of them as well as never users i.e. 123 (75%) of them. The periodontal pocket of 4-5 mm was found in 27 (37.5%) and pocket of 6 mm or more in 6 (8.3%) of the current users. Loss of attachment of 4-5 mm was found in 30 (41.7%) current users and 6 (3.7%) never users, 6-8 mm was found in 5 (6.9%) current users and 5 (3.0%) never users (Table 3).

**Table 3:** Periodontal status (CPI modified) among different groups of tobacco users.

Oral findings		Current users	Ever users	Never users
		n (%)	n (%)	n (%)
Gingival bleeding	Present	34 (47.2)	10 (52.6)	41 (25.0)
	Absent	38 (52.8)	9 (47.4)	123 (75.0)
Periodontal pocket	Absent	39 (54.2)	13 (68.4)	139 (84.8)
	Pocket 4 -5 mm	27 (37.5)	6 (31.6)	21 (12.8)
	Pocket 6 mm or more	6 (8.3)	0 (0.0)	4 (2.4)
Loss of attachment	0 - 3 mm	35 (48.6)	12 (63.2)	151 (92.1)
	4 - 5 mm	30 (41.7)	2 (10.5)	6 (3.7)
	6 - 8 mm	5 (6.9)	3 (15.8)	5 (3.0)
	9 - 11 mm	2 (2.8)	2 (10.5)	2 (1.2)
	12 mm or more	0 (0.0)	0 (0.0)	0 (0.0)

## DISCUSSION

Both smoked and smokeless forms of tobacco constitute major public health problems. The present study shows that mean decayed and loss of a tooth is found to be increased in individuals consuming tobacco in either form. It shows that 28.2% of the total participants were current users of which 34.7% used smoked form, 44.4% used smokeless form and 20.8% used both forms of tobacco. A study by Agbor, et al.^7^ has shown the prevalence of smokeless tobacco to be 52.7%. The majority of the current users i.e. 58.3% belonged to the age group of 35-64 years and never users i.e. 64.6% belonged to the age group of 18-34 years. Also, in our study 77.8% of the current users were males. The findings are similar to a study by Bidhyasagar, et al.^8^ showed that non-users were significantly more among the age group of 25-34 years and more in males. The most commonly occurring oral health problems associated with smoking were found to be a gingival recession, tooth wear, tooth loss followed by caries.^7^ Mean number of mobile teeth and missing teeth were found to be significantly higher among the non-users.^8^

Mean decayed teeth value was found to be more in smokers as compared to non-smokers in the current study as well as supported by other studies.^7-9^ Higher mean DMFT and CPI scores were also recorded in the case of smokers than non-smokers in a study by Akaji and Folaranmi.^10^ There are very few studies showing a direct etiological relationship of tobacco for dental caries but studies have shown higher counts of Lactobacillus and Streptococcus mutans.^11^

Higher mean Community Periodontal Index (CPI) scores were observed in smokers than in non-smokers.^10^ Studies by Reddy, et al. showed the prevalence of smoking to be 72.5% with a mean DMFT of 5.26 and the majority of them having a CPI score of 2. The current study also showed better gingival and periodontal health with the majority of never users showing an absence of gingival bleeding. Also, the majority of never users showed an absence of a periodontal pocket and the majority of current users showed a loss of attachment of 4-5mm. Studies have shown tobacco to be an independent risk factor for periodontal diseases whose severity increases with the increase in frequency. It is also found to be associated with the increased pocket formation and loss of attachment.^11,12^ Therefore, these findings show that smoking has a significant effect on dental caries, tooth loss as well as periodontal health.

The major limitation of this study is the study participants are limited to those visiting dental OPDs so the results cannot be generalized. Also, under-reporting of the tobacco habits by the study participants may also take place.

## CONCLUSIONS

The prevalence of tobacco use among our study participants is similar in comparison to that of the general population. The current study showed a greater prevalence of the use of a smokeless form of tobacco than the smoked form of tobacco. And the current users showed a greater mean decayed score as well as DMFT score. Besides this, current users showed a greater prevalence of gingival bleeding, periodontal pocket and loss of attachment.
